# ALiEM Blog and Podcast Watch: Toxicology

**DOI:** 10.5811/westjem.2017.6.33952

**Published:** 2017-09-11

**Authors:** Fareen Zaver, Michael Craddick, Audrey Sanford, Nana Sefa, George Hughes, Michelle Lin

**Affiliations:** * University of Calgary, Department of Emergency Medicine, Calgary, Alberta, Canada; † University of Illinois College of Medicine at Peoria, Department of Emergency Medicine, Peoria, Illinois; ‡ University of Illinois at Chicago, Department of Emergency Medicine, Chicago, Illinois; § William Beaumont Hospital, Department of Emergency Medicine, El Paso Texas; ¶ University of Illinois at Chicago, Department of Emergency Medicine, Chicago, Illinois; || University of California San Francisco, Department of Emergency Medicine, San Francisco, California

## Abstract

**Introduction:**

The WestJEM Blog and Podcast Watch presents high-quality open-access educational blogs and podcasts in emergency medicine based on the ongoing Academic Life in Emergency Medicine (ALiEM) Approved Instructional Resources (AIR) and AIR-Professional (Pro) series. Both series critically appraise open-access educational blogs and podcasts in EM using an objective scoring instrument. This installment of the blog and podcast watch series curated and scored relevant posts in the specific topic of toxicology emergencies from the AIR-Pro Series.

**Methods:**

The AIR-Pro Series is a continuously building curriculum covering a new subject area every two months. For each area, eight EM chief residents identify 3–5 advanced clinical questions. Using FOAMsearch.net and FOAMSearcher to search blogs and podcasts, relevant posts are scored by eight reviewers from the AIR-Pro editorial board, which is comprised of EM faculty and chief residents at various institutions across North America. The scoring instrument contains five measurement outcomes based on seven-point Likert scales: recency, accuracy, educational utility, evidence based, and references. The AIR-Pro label is awarded to posts with a score of ≥28 (out of 35) points. An “honorable mention” label is awarded if board members collectively felt that the blogs were valuable and the scores were > 25.

**Results:**

A total of 31 blog posts and podcasts were included. Key educational pearls from the six high-quality AIR-Pro posts and four honorable mentions are summarized.

**Conclusion:**

The WestJEM ALiEM Blog and Podcast Watch series is based on the AIR and AIR-Pro Series, which attempts to identify high-quality educational content on open-access blogs and podcasts. This series provides an expert-based, crowdsourced approach towards critically appraising educational social media content for EM clinicians. This installment focuses on toxicology emergencies.

## INTRODUCTION

Despite the rapid rise in social media educational content on blogs and podcasts, especially in emergency medicine (EM),^1^ there has only been preliminary progress in helping educators and learners identify quality resources.^2^–^4^ In 2008 the Accreditation Council for Graduate Medical Education endorsed a decrease in synchronous conference experiences for EM residency programs by up to 20% in exchange for asynchronous learning, termed individualized interactive instruction (III).^5^ Residency programs, however, were often unsure how to identify quality online resources specifically for asynchronous learning and III credit.

To address this need, the Approved Instructional Resources (AIR) Series^6^ and AIR-Professional (Pro) Series were created in 2014 and 2015, respectively, by Academic Life in Emergency Medicine (ALiEM) to help EM residency programs identify quality online content specifically on social media. Using an expert-based, crowdsourced approach, these two programs identify trustworthy, high-quality educational blog and podcast content. The intended audience for the AIR series is EM junior residents, and for the AIR-Pro Series is the EM advanced practitioner. This blog and podcast watch series on *WestJEM* presents annotated summaries from the AIR and AIR-Pro Series.

This installment from the AIR-Pro Series summarizes the best scoring social media educational resources on specific topics within toxicology emergencies.

## METHODS

### Question Identification

The AIR-Pro Series is a continuously building curriculum covering a new subject area every two months. For each area, eight EM chief residents from different U.S. residency programs on the ALiEM-Pro editorial board identify 3–5 focused, advanced-level clinical queries within the featured subject area. The topics for this installment included the following:

1. Flumazenil in benzodiazepine overdose
2. Acetaminophen – drawing and timing of levels
3. Opioid overdoses
4. Acetaminophen toxicity related to liver transplant
5. Salicylates and hemodialysis

### Inclusion and exclusion criteria

All available blog posts and podcasts on these five topics were identified using two custom EM search engines: FOAMsearch.net and FOAMSearcher. Blog posts and podcasts written in English and identified by key search terms were included for our scoring by our expert panel. Journal articles were excluded from the list.

### Scoring

Extracted posts were scored by eight reviewers from the AIR-Pro editorial board, which is comprised of EM core faculty and chief residents from various U.S. institutions. The eight reviewers included five chief residents from the AIR-Pro editorial board as well as three EM faculty educators. The scoring instrument contains five measurement outcomes using seven-point Likert scales: recency, accuracy, educational utility, evidence based, and references (Table 1).

### Data Analysis

An AIR-Pro endorsement is given to posts with a score of ≥28 (out of 35) points. Depending on the redundancy of the highest scoring posts, the best of these are then selected to address each pre-selected topic. An “honorable mention” label is also given to posts specifically felt to be worthwhile, accurate, unbiased, and educationally valuable for advanced clinicians by consensus of the AIR-Pro board. These posts must have scored ≥25 (out of 35) points.

## RESULTS

A total of 31 blog posts and podcasts were initially included. Key educational pearls from the six high-quality, AIR-Pro posts and four honorable mentions are summarized (Table 2).

### AIR-Pro Content

#### 1. Awad N. Flumazenil: Friend or Foe? EM PharmMD. (November 7, 2013) http://empharmd.blogspot.ca/2013/11/flumazenil-friend-or-foe.html

This blog post discusses the incidence of seizures associated with the use of flumazenil in benzodiazepine overdose. It provides evidence that questions the long-held belief regarding the risk of seizures associated with the use of flumazenil.

##### Take home points

The post acknowledges that earlier studies documented a 13% incidence of seizures associated with the use of flumazenil; however, a number of recent studies put that rate at about 1%. Although the true incidence of seizures with the use of flumazenil cannot be precisely ascertained, it should be used with caution. The use of flumazenil is warranted for the following specific emergent situations in non-chronic benzodiazepine users: pediatric ingestions, iatrogenic toxicity, and a paradoxical response associated with a pure benzodiazepine overdose.

#### 2. Hayes B. Utility of Pre-4-Hour Acetaminophen Levels in Acute Overdose. *ALiEM*. (August 5th, 2015) https://www.aliem.com/2015/utility-of-pre-4-hour-acetaminophen-levels-in-overdoses/

Through a review of multiple studies addressing the utility of pre-four hour levels, the timing of acetaminophen levels are examined including the interpretation of these levels.

##### Take-home points

Undetectable levels drawn after one hour of ingestion suggest that it is unlikely that the four-hour level will be clinically significant. The Rumack-Matthew nomogram, however, can only be used with an adequate negative predictive value when acetaminophen levels are drawn four hours after ingestion. Emergency physicians should continue to aim to draw levels after four hours of ingestion, but especially within seven hours if possible to ensure timely treatment with N-acetylcysteine if necessary.

#### 3. Carley S. Opiate Overdose in the ED. *St.Emlyn’s*. (February 27, 2015) http://stemlynsblog.org/opiate-overdose-in-the-ed-st-emlyns/

This 23-minute podcast, with a subsequent blog summary, covers the approach to patients with an opioid overdose. It emphasizes cautious reversal and continuous monitoring to ensure patient safety.

##### Take home points

Consider opioid ingestion in patients with toxidrome findings of miosis, central nervous system depression, respiratory depression, and consequences of prolonged hypoxia (seizures, dysrhythmias, brain injury). Do not give high doses of naloxone out of concern for precipitating withdrawal, unless the patient is in cardiac arrest. Naloxone has a shorter half-life than most long-acting opioids and will often need to be re-dosed. This is especially relevant in the patient who received one dose with significant response who is threatening to leave the department. Prior to discharge or admission it is also important to evaluate a patient for a possible intentional overdose requiring psychiatric evaluation, rhabdomyolysis or compartment syndrome due to prolonged unconsciousness, as well as for substance-abuse referral.

#### 4. Cohn B, Schwarz E. Treat and Release vs. Observation After Naloxone for Opioid Overdose. *EMJ Club Podcast 17*. (November 24, 2014) http://emjclub.com/podcast/observation-after-naloxone

This 18-minute podcast uses a clinical case scenario to highlight the disposition considerations in the case of patients who overdose on opioids and received naloxone. Through a PubMed search, the podcast uses a journal club approach to analyze four articles on the topic.

##### Take home points

The literature supports a “treat and release” strategy for a specific set of patients who have overdosed on opioids. These patients must return to their pre-overdose baseline, be hemodynamically stable and alert, and understand the risks versus benefits of their medical condition prior to discharge. This strategy has not been tested in an overdose of long-acting opioids such as methadone. Thus, caution should be applied in its use in this scenario since rebound symptoms are common with long-acting opioids.

#### 5. Nickson C. Liver Transplantation for Paracetamol Toxicity. *Life in the FastLane*. (April 30, 2016) http://lifeinthefastlane.com/ccc/liver-transplantation-for-paracetamol-toxicity

This post is an overview of liver failure in the setting of acetaminophen (paracetamol) toxicity. Nickson outlines the details for identification of possible transplant candidates, utility of the King’s College Criteria and research thus far on long-term outcomes of acute liver failure from toxicity.

##### Take-home points

Keep a low threshold for transferring patients to a hepatobiliary transplant center for possible transplant if there are any signs to suggest severe end-organ damage. The King’s College Criteria is the most commonly used tool for identifying transplant candidates. More recent studies suggest that survival rates without a liver transplant is improving and questions the utility of liver transplant in most cases.

King’s College Criteria:

- pH < 7.3, or
- All below (within 24-hr period):
- INR > 6 (PT > 100s)
- Cr > 300 mmol/L
- grade III or IV encephalopathy

#### 6. Fontes K. 5 Tips in Managing Acute Salicylate Poisoning. *ALiEM.* (November 4, 2013) https://www.aliem.com/2013/5-tips-in-managing-acute-salicylate-poisoning/

This blog post highlights several key clinical pearls in managing acute salicylate poisoning. Main discussion points include serum levels and concentrations, treatment with alkalinization, and indications for hemodialysis.

##### Take home points

Trending a patient’s serum salicylate levels is more important than a single value level. Furthermore, acidosis correlates with severity of illness. The goal of treatment is to maintain a serum pH of 7.50–7.55 by adding three ampules (50 mL each) of 8.4% sodium bicarbonate to one liter of 5% dextrose in water. Avoid intubation if possible, but if necessary give sodium bicarbonate prior and hyperventilate the patient after to maintain compensatory respiratory alkalosis. Emergent hemodialysis should be seriously considered for patients with serum salicylate levels >100 mg/dL, as well as for patients with any salicylate level plus severe symptoms such as central nervous system dysfunction, renal failure, cerebral/pulmonary edema, or unexplained acid-based disturbance.

#### 7. Nickson C. Paracetamol/Acetaminophen Overdose. *Life in the FastLane.* (Sept 3, 2010) http://lifeinthefastlane.com/paracetamol-overdose/

This blog post summarizes the key pearls in the epidemiology, identification and management of acetaminophen overdose.

##### Take home points

Paracetamol/acetaminophen (APAP) is the most common medication taken in overdose and the number one cause of acute liver failure in the U.S. APAP toxicity is typically thought to occur in four stages although these symptoms and timeline are not always consistent.

- Stage 1 (0–24 hr): Preclinical stage — Nonspecific symptoms
- Stage 2 (24–72 hr): Onset of liver injury — Nausea and vomiting, right upper quadrant pain, abnormal liver function tests, elevated lactate and creatinine
- Stage 3 (72–96 hr): Maximal hepatotoxicity — Liver failure, renal failure, coagulopathy, hypoglycemia, encephalopathy
- Stage 4 (>5 days): Recovery phase if the patient survives — Resolution of hepatotoxicity

Activated charcoal was previously used in the setting of acute ingestion, but is much less effective after 1–2 hours after ingestion. N-acetylcysteine (NAC) is now the standard treatment. NAC promotes the non-toxic metabolism of acetaminophen and should be started within eight hours of ingestion for maximum efficacy. If the patient presents after eight hours from ingestion with a significant risk for toxicity, it is reasonable to start IV NAC without an acetaminophen level as the risks of waiting outweigh the mild side effects of the drug such as flushing or a rash.

#### 8. Hayes B. Tricks of the Trade: Naloxone Dilution for Opioid Overdose. *ALiEM.* (Nov 17, 2014) https://www.aliem.com/2014/trick-trade-naloxone-dilution/

This blog post discusses how to dilute naloxone to provide better titration in reversing the signs and symptoms from an opioid overdose, without inducing symptoms of opioid withdrawal.

##### Take Home Points

In a 10-mL syringe, combine 9 mL of sterile normal saline and 1 mL of 0.4 mg/mL naloxone. This results in a 10 mL solution of 0.04 mg/mL naloxone. Label the syringe and administer 1–2 mL (equal to 0.04–0.08 mg) of naloxone every 60 seconds to achieve the desired clinical state.

#### 9. Nickson C. Paracetamol. *Life in the FastLane.* (2015) http://lifeinthefastlane.com/tox-library/toxicant/analgesia-and-anti-inflammatories/paracetamol/

This blog post is a review of the approach to acetaminophen (paracetamol) ingestion given different and complex ingestion scenarios.

##### Take Home Points

The toxic dose of acetaminophen in adults is >10 g or >200 mg/kg in 24 hours. The Rumack-Matthew nomogram is only validated for a single ingestion of acetaminophen; it is unreliable for multiple ingestions, delayed presentation, or modified release preparations. If a patient presents in the 8–24 hour period after the ingestion, obtain acetaminophen levels and liver enzymes to guide management, even if the patient is asymptomatic. For massive ingestions >30 g, higher doses of NAC may be necessary and will require expert consultation.

#### 10. Bright J. Pearls and Pitfalls of Salicylate Toxicity in the Emergency Department. *emDoc*s. (October 15, 2015) http://www.emdocs.net/pearls-and-pitfalls-of-salicylate-toxicity-in-the-emergency-department/

This blog post discusses the acute salicylate toxicity, specifically focusing on the optimization of fluid, electrolytes, and acid-base management.

##### Take Home Points

A diagnostic pitfall is to rely solely on down trending salicylate levels alone. Down trending levels alone are not always reassuring for the patient’s clinical course; be sure to monitor for signs of central nervous system (CNS) toxicity. Regarding management, patients are often hypovolemic, hypokalemic, and acidotic. Thus, ensure adequate fluid resuscitation, replace potassium levels to a goal of >4 mEq/L, and correct acidemia with sodium bicarbonate in dextrose 5% water (D5W) to a goal serum pH 7.45–7.55 to enhance elimination. Serum glucose levels should be maintained >150 mg/dL to prevent CNS-related hypoglycemia. If intubation is necessary, maintain pre-intubation minute ventilation to avoid worsening acidemia. Do not delay hemodialysis for those with serum levels >100 mg/dL or signs of significant toxicity.

## CONCLUSION

The *WestJEM* Blog and Podcast Watch series serves to identify educational quality blogs and podcasts for EM clinicians through its expert panel using an objective scoring instrument. These social media resources are currently curated in the ALiEM AIR and AIR-Pro Series, originally created to address EM residency needs. These resources are herein shared and summarized to help clinicians filter the rapidly published multitude of blog posts and podcasts. While these lists are by no means a comprehensive analysis of the entire Internet for these topics, this series provides a post-publication accreditation and curation of recent, online content to identify and recommend high-quality educational social media content for the EM clinician.

## Figures and Tables

**Table 1:** AIR-Pro scoring instrument for blog and podcast content (maximum score = 35 points).

Tier 1: recency	Score	Tier 2: content accuracy	Score	Tier 3: educational utility	Score	Tier 4: evidence based- medicine	Score	Tier 5: referenced	Score
When was the blog post or podcast published?		Do you have any concerns about the accuracy of the data presented or conclusions of this article?		Are there useful educational pearls in this article for senior residents?		Does this article reflect evidence-based medicine (EBM) and thus lack bias?		Are the authors and literature clearly cited?	
≥6 years ago or unknown	1	Yes, many concerns from many inaccuracies	1	Low value: No valuable pearls	1	Not EBM based, only expert opinion	1	No	1
5–6 years ago	2		2		2		2		2
4–5 years ago	3	Yes, a major concern about few inaccuracies	3	Yes, but there are only a few (1–2) valuable or multiple (>=3) less-valuable educational pearls	3	Minimally EBM based	3		3
3–4 years ago	4		4		4		4	Yes, authors and general references are listed (but no in-line references)	4
2–3 years ago	5	Minimal concerns over minor inaccuracies	5	Yes, there are several (>=3) valuable educational pearls, or a few (1–2) KEY educational pearls that every resident should know before graduating	5	Mostly EBM based	5		5
1–2 years ago	6		6		6		6		6
<1 year ago	7	No concerns over inaccuracies	7	Yes, there are multiple KEY educational pearls that residents should know before graduating	7	Yes exclusively EBM based (unbiased)	7	Yes, authors and in-line references are provided	7

**Table 2:** Blog posts and podcasts receiving an AIR-Pro endorsement or honorable mention on the topic of toxicology.

Article title	Authors	Date	Title	Website URL
Flumazenil: Friend or Foe?	Nadia Awad	Nov 7, 2013	AIR-PRO	http://empharmd.blogspot.ca/2013/11/flumazenil-friend-or-foe.html
Utility of Pre-4-Hour Acetaminophen Levels in Acute Overdose	Bryan Hayes	Aug 5, 2015	AIR-PRO	https://www.aliem.com/2015/utility-of-pre-4-hour-acetaminophen-levels-in-overdoses/
Opiate Overdose in the ED	Simon Carley	Feb 27, 2015	AIR-PRO	http://stemlynsblog.org/opiate-overdose-in-the-ed-st-emlyns/
Treat and Release vs. Observation After Naloxone for Opioid Overdose	EMJ Club	Nov 24, 2014	AIR-PRO	http://www.foamem.com/2014/11/24/treat-and-release-vs-observation-after-naloxone-for-opioid-overdose/
Liver Transplantation for Paracetamol Toxicity	Chris Nickson	April 30, 2016	AIR-PRO	http://lifeinthefastlane.com/ccc/liver-transplantation-for-paracetamol-toxicity/
5 Tips in Managing Acute Salicylate Poisoning	Kristin Fontes	Nov 4, 2013	AIR-PRO	https://www.aliem.com/2013/5-tips-in-managing-acute-salicylate-poisoning/
Paracetamol/Acetaminophen Overdose	Chris Nickson	Sept 3, 2010	Honorable Mention	http://lifeinthefastlane.com/paracetamol-overdose/
Tricks of the Trade: Naloxone Dilution for Opioid Overdose	Bryan Hayes	Nov 17, 2014	Honorable Mention	https://www.aliem.com/2014/trick-trade-naloxone-dilution/
Paracetamol	Chris Nickson	2015	Honorable Mention	http://lifeinthefastlane.com/tox-library/toxicant/analgesia-and-anti-inflammatories/paracetamol/
Pearls and Pitfalls of Salicylate Toxicity in the Emergency Department	Justin Bright	Oct 13, 2015	Honorable Mention	http://www.emdocs.net/pearls-and-pitfalls-of-salicylate-toxicity-in-the-emergency-department/
